# Early Motor Interventions in Infants and Young Children: A Comprehensive Scoping Review

**DOI:** 10.3390/children13050644

**Published:** 2026-05-04

**Authors:** Sophia Charitou, Emmanouil Skordilis

**Affiliations:** School of Physical Education and Sport Science, National and Kapodistrian University of Athens, 17237 Athens, Greece; eskord@phed.uoa.gr

**Keywords:** early motor intervention, motor development, infancy, neurodevelopment, cerebral palsy, developmental delay, early childhood

## Abstract

**Highlights:**

**What are the main findings?**

- Effective early motor intervention programs are characterized by early initiation, active engagement, task-specific training, sufficient intensity, and caregiver involvement.
- Early motor interventions support not only motor development but also broader domains, including cognitive (e.g., executive function) and social-emotional development.

**What are the implications of the main findings?**

- Early motor interventions should be recognized as multidimensional strategies that influence multiple developmental domains beyond physical outcomes.
- Interventions that are timely, intensive, individualized, and embedded within the child’s natural environment are associated with more favorable developmental outcomes.

**Abstract:**

Background: Early motor milestones play a critical role in shaping developmental trajectories across motor, cognitive, social, and functional domains. Increasing evidence indicates that motor competence facilitates environmental exploration, learning opportunities, and social engagement during infancy and early childhood. Methods: The present scoping review aimed to map and synthesize the existing evidence on early motor interventions in children aged 0–6 years across diverse pediatric populations. A comprehensive literature search was conducted across PubMed, Scopus, and Web of Science. Studies were selected based on predefined inclusion criteria, and data were extracted and synthesized using a descriptive and thematic approach. Results: A total of 30 studies were included, encompassing a wide range of populations, including preterm infants, children at risk of cerebral palsy, and typically developing children. Across studies, early motor interventions were associated with improvements in motor outcomes and, in many cases, broader developmental domains such as cognition and social interaction. Intervention effectiveness appeared to be influenced by factors such as timing, intensity, task specificity, and caregiver involvement. Conclusions: The review provides a cross-population synthesis of early motor interventions and proposes a conceptual framework that integrates shared mechanisms underlying effective intervention across diverse pediatric groups. This approach offers a more unified understanding of how early motor interventions influence developmental trajectories beyond diagnosis-specific perspectives.

## 1. Introduction

### 1.1. Early Development

Early development represents a sensitive period characterized by rapid neurodevelopmental changes. Infancy and early childhood represent periods of rapid neural reorganization characterized by accelerated plasticity and a dynamic interaction between biological maturation and experience-dependent environmental influences [1,2]. During these sensitive windows, neural circuits underlying movement, perception, cognition, and social behavior are constantly formed through environmental input (sensory experiences and practiced behavior). Thus, development is complex and non-linear, since several domains work together in a reciprocal process [3]. Motor development is increasingly recognized as a key driver of the above processes. Motor skill acquisition provides the foundation for exploration, object manipulation, and social engagement and is not a peripheral end product of physical maturation. In turn, engagement in motor activities supports the collection of sensory inputs that form the neural circuitry and eventually the development of cognitive skills [4]. Previous research has demonstrated a significant association between motor skills and cognitive/social development in preschoolers, suggesting that motor development is a vital vehicle through which broader developmental pathways are developed [1]. Both longitudinal and cross-sectional research, has also established that the attainment of early developmental motor skills predicts later functional performance, such as involvement in school, physical activity, and school preparedness [5,6]. The above findings have accumulated research attention in developmental science, suggesting that the development of motor abilities is now the driving force in shaping developmental potential, rather than a second or a tertiary reflection.

### 1.2. Importance of Early Motor Intervention

Since the acquisition of motor skills is fundamental to development, early motor interventions have become an important aspect of pediatric care and research. Early motor intervention refers to structured/semi-structured programs designed to promote the acquisition of motor skills in infants and toddlers, especially those at high risk of being developmentally delayed [2,3]. Such programs may be delivered in hospitals, homes, childcare centers and/or community centers and can be provided by therapists, family members, educators/team players, specialists or professional groups. Further, approaches of intervention have changed considerably during recent decades. Traditional approaches, for example, aimed towards the normalization of movement patterns with therapist-centered strategies. Current strategies however focus on active child involvement, outcome-oriented activities, enriched environment, and caregiver participation [7]. The development of current strategies is consistent with neuroscientific evidence showing that experience-based neural plasticity reaches its peak in infancy, which is a time of great chance to start interventions [8,9]. Evidence suggests that early developmental programs can improve motor and cognitive outcomes in at-risk infants occur via systematic reviews, especially where they are intensive, individualized, and functionally tailored [3,10]. Active motor engagement leads to better outcomes than passive stimulation [11] and it emphasizes the value of child-centeredness in learning.

Recent evidence further strengthens the rationale for early motor intervention by highlighting its influence on early neurodevelopmental processes. A systematic review demonstrated that early intervention may positively affect spontaneous motor patterns, particularly during the fidgety period, which is closely associated with later neurodevelopmental outcomes [12]. In addition, caregiver-mediated approaches have been shown to improve motor development while enhancing caregiver engagement and adherence to intervention strategies. Experimental evidence also supports the effectiveness of home-based, post-discharge interventions in preterm infants, demonstrating improvements in both motor development and physical growth [7,13]. These findings collectively reinforce the importance of early, active, and family-centered intervention models.

### 1.3. Populations at Developmental Risk

Early motor interventions are particularly pertinent for high-risk populations experiencing a developmental delay. These populations include preterm infants, children with neuro-developmental conditions such as cerebral palsy, developmental coordination disorders, autism spectrum disorders, and congenital conditions [1,10,14,15,16]. Further many preterm infants have been widely studied in intervention research. While advances in neonatal treatment with improved survival have made many preterm infants more comfortable in the womb, many remain at risk of motor, cognitive, and behavioral difficulties. In this regard, studies showed that earlier, neonatal or soon after hospital discharge-implemented developmental programs decrease the risk of neurodevelopmental disability [1,17]. Likewise, children with early neurological disease or congenital disease may undergo motor delays that are associated with increased developmental disability over time. It is therefore imperative to identify and intervene early to maximize developmental outcomes. According to clinical guidelines, infants with high risk of cerebral palsy should receive early and focused intervention as early as possible, using goal directed treatments when risk is recognized rather than waiting for a formal diagnosis [18]. Children with developmental coordination disorder form another important group of research attention. Motor coordination deficits can interfere with everyday life, schooling, and engagement. Early identification combined with motor-based interventions can achieve improvements in functional abilities and mitigate long-term participation constraints [14,15]. Recent evidence also suggested that children with autism spectrum disorder benefit from early motor-related interventions. Evidence of motor skills achievements from motor training programs has been reported in the autistic population that may lead to enhanced social activity and well-rounded social engagement [11]. Lastly, children with congenital medical conditions such as congenital heart disease may exhibit motor delay as a result of long hospitalization, operations and reduced mobility. Early intervention studies on these populations have reported promising but minimal benefit [10].

### 1.4. Conceptual Shift in Intervention Philosophy

Pediatric rehabilitation has undergone an important paradigm shift over the past twenty years. Older treatments had been mainly focused upon impairments and attempted to ameliorate atypical movement patterns or abnormal muscle tone through therapist-guided methods of normalization rather than function. The current models are more focused on training specific skills, participating in daily life, or integrating the intervention within authentic experiences. Development thus evolves as a phenomenon beyond clinical contexts. Ecological and family-based models focus on interaction with caregivers and the embedding of intervention in their everyday lives, enabling practice, and skill generalization [7,13]. The above shift is also supported by systematic reviews. Active involvement, meaningful activities and context related programs can often be more effective than passive or decontextualizing interventions [9,19]. Parent-mediated interventions are also associated with improved developmental results, emphasizing the role that caregivers play in early intervention [9,19]. The positive involvement of caregivers and adults in general is consistent with recent clinical work. Previous reactions only targeted corrective measures to deficits, whereas current intervention systems strive with a view to involve significant others, increase participation, independence, and full developmental competence. These approaches fit nicely with frameworks that emphasize a philosophy for the enhancement of functional outcomes and quality of life.

### 1.5. The Need for Evidence Mapping

Although research on early motor intervention has grown significantly, the literature is still heterogeneous. Studies differ significantly for purposes of target populations, intervention strategies, intensity and duration, endpoint measures and methods. A large number of systematic reviews focus on specific diagnoses or types of interventions, and there are limited opportunities for cross-cutting comparisons among populations. In addition, the nature of the study design and reporting complicates comparisons across interventions. Variation in dosage, assessment instruments and follow-up timings may alter interpretation of findings. Methodological inconsistency has been an essential limitation of the literature for decades, with reviews [9,11,13] frequently stating methodological inconsistency is a significant limitation within the field. Thus, a scoping review is a suitable approach to synthesize the present body of research work. Scoping reviews in contrast to conventional systematic reviews—which usually focus on narrowly oriented questions—attempt to map, as described in our definition, the breadth and characteristics of the evidence across a domain. It also helps in identifying patterns, gaps, and research priorities among various study designs and across populations, across a wide range of studies.

### 1.6. Objectives

The objectives of the present scoping review were: 1. To map the research on early motor intervention for infants and young children. 2. To summarize intervention characteristics across populations and settings. 3. To compile reported outcomes across developmental domains. 4. To find methodological limitations in literature. 5. To suggest future research and applications in clinical settings.

By synthesizing findings across diverse pediatric populations, the present review provides an integrative perspective on early motor interventions and highlights shared principles of effective intervention strategies. Unlike previous reviews that focus on specific diagnostic groups, the present study adopts a cross-population perspective to identify shared mechanisms underlying effective early motor interventions. Importantly, this review proposes a cross-population conceptual framework that integrates common principles of effective intervention across diverse pediatric populations, providing a structured understanding of how motor interventions influence developmental trajectories.

## 2. Materials and Methods

### 2.1. Study Design

A scoping review methodology was employed to account for the wide range and diversity of available evidence. This approach is particularly appropriate for research areas characterized by conceptual complexity, heterogeneity in study design, and emerging research questions. The review followed the methodological framework proposed by Peters et al. [20] and was conducted in accordance with the PRISMA Extension for Scoping Reviews (PRISMA-ScR) guidelines [21].

### 2.2. Search Strategy

A comprehensive literature search was conducted across PubMed, Scopus, and Web of Science using combinations of keywords and Boolean operators related to early motor intervention and child development.

The search strategy included the following terms: (“early intervention” OR “early motor intervention” OR “motor development” OR “motor skills”) AND (“infants” OR “children” OR “early childhood”) AND (“intervention” OR “therapy” OR “training”).

Searches were limited to peer-reviewed articles published in English between 2005 and 2025. Reference lists of included studies were also screened to identify additional relevant publications. The search strategy was intentionally focused to ensure conceptual clarity and relevance to early motor interventions. The number of included studies reflects the application of strict inclusion criteria, prioritizing studies that specifically examined early motor interventions in children aged 0–6 years with clearly defined developmental outcomes.

### 2.3. Eligibility Criteria

Eligible studies met the following inclusion criteria:

- included children aged 0–6 years
- examined interventions targeting motor development
- reported motor or broader developmental outcomes
- were published in peer-reviewed journals

Eligible study designs included systematic reviews, meta-analyses, randomized controlled trials, pilot studies, clinical guidelines, and scoping reviews. No restrictions were placed on geographic location or intervention setting.

### 2.4. Study Selection

All identified records were imported into a reference management system and duplicates were removed. Two independent reviewers screened titles and abstracts based on predefined eligibility criteria. Full-text articles were subsequently assessed for eligibility. Disagreements between reviewers were resolved through discussion until consensus was reached.

### 2.5. Data Extraction

The following information was extracted from each study:

- authors and year of publication
- study design
- participant characteristics
- type of intervention
- setting
- outcome domains
- main findings
- limitations reported by the authors

Data extraction was performed independently by two reviewers using a standardized data charting form. Disagreements were resolved through discussion until consensus was reached.

### 2.6. Sources of Evidence

**Figure 1:**
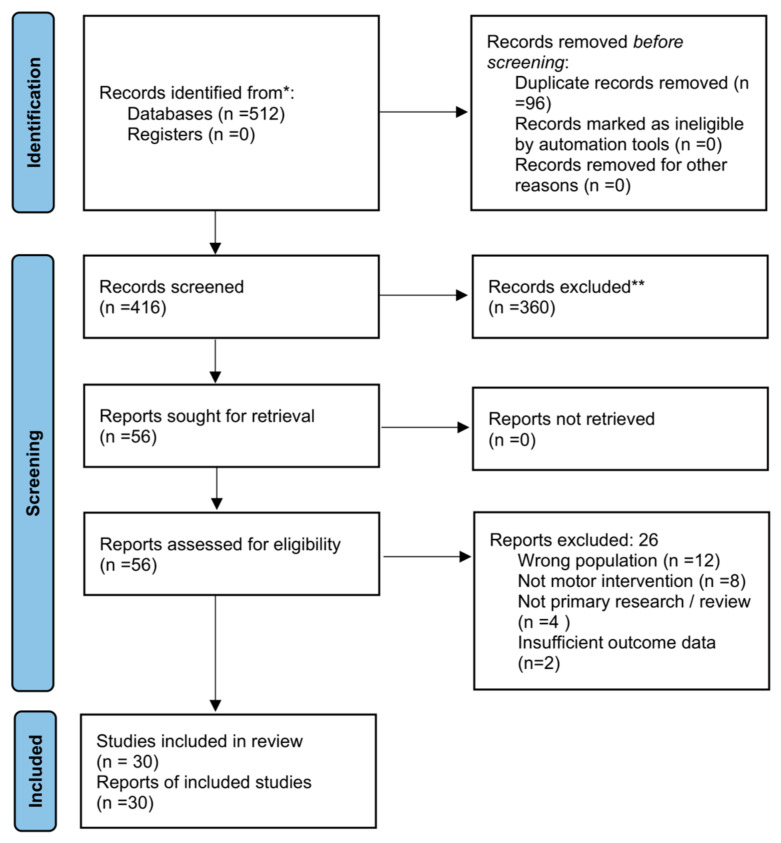
PRISMA-ScR flow diagram illustrating the study selection process.

The dataset comprised thirty peer-reviewed publications published between 2005 and 2025. These studies represent a broad spectrum of populations, intervention approaches, and methodological designs. Literature was identified through systematic searches in major academic databases, including PubMed, Scopus, and Web of Science. Google Scholar was additionally used to screen reference lists and identify potentially relevant studies not captured in database searches. The study selection process is presented in the PRISMA-ScR flow diagram (Figure 1).

### 2.7. Risk of Bias

Consistent with scoping review methodology, a formal risk-of-bias assessment was not conducted. This approach allows for a broad mapping of available evidence; however, it may limit conclusions regarding the strength of the evidence.

### 2.8. Synthesis of Data

Data were synthesized using descriptive summaries and thematic analysis. Studies were grouped according to population, intervention characteristics, and outcome domains. Patterns across studies were identified and integrated to provide a comprehensive overview of the evidence. Consistent with the methodological framework of scoping reviews, the aim was to map the breadth of available evidence rather than to evaluate study quality.

## 3. Results

### 3.1. Overview of Included Evidence

Types of studies: systematic reviews, meta-analyses, randomized trials, pilot studies, clinical guidelines, and scoping reviews. The strength of evidence is indicative of ongoing interest in early motor intervention, especially in a number of pediatric populations. Many of these studies focused on high-risk populations, particularly preterm infants and children who are at risk for cerebral palsy. However, studies of typically developing children can illustrate the value of early motor intervention as a therapeutic and preventive method [22,23,24]. Detailed characteristics of the included studies are presented in Supplementary Table S1.

### 3.2. Preterm Infants

Preterm birth has been linked to higher risks of motor delay, cognitive impairment, and behavioral issues. Comprehensive review findings indicated that developmental interventions carried out during the neonatal period and just days following discharge can reduce risk for neurodevelopmental disorder in premature infants [13,25,26]. Active motor-centered interventions appeared particularly successful. Programs that promote the development of independent movements in babies are more likely to be effective than passive developmental programs [27]. Caregiver involvement was also central: early interventions that trained the capabilities of parents and were delivered very early in life and at home can enhance neurodevelopmental trajectories and sustainability of gains [28]. Focused studies of locomotor training further validated feasibility and effectiveness. Controlled trials of early crawling instructions revealed effective locomotor development of very preterm infants [29]. In total, the above findings contribute to a potential implication of early onset in the premature child and suggest that to ameliorate effects, both active participation and caretakers’ participation are important.

### 3.3. Children Developing at Risk for Cerebral Palsy

Infants with or at risk for CP have been the most studied population in early intervention research. Identification of early onset of neurological risk could ensure that intervention may be initiated at a period when neuroplasticity is higher and supportive of the developmental paths attained through training. Indeed, systematic reviews have shown that early motor interventions have beneficial effects on motor function, daily activities and participation in early stages of CP in infants who are either affected or at risk of CP [9,30]. International clinical guidelines recommend prevention as early as risk is recognized and without waiting for an unequivocal diagnosis. Targeted and task-specific training is recommended by these guidelines for 0–2-year-old babies [31]. Active movement therapy has produced positive results in clinical trials, serving as an early childhood development intervention by promoting self-initiated movement. It is also recognized as a facilitator of development [27]. Additional proof suggests that enriched motor training experience, and practice, are required to enhance skills and adaptive neural reorganization [2]. Encouraging caregivers to provide services at home (home-based programs interventions) was highly effective as well. Children exposed to intervention-based practices in their usual routine demonstrated motor skill development and general developmental benefits [7,19]. RCTs showed that structured early interventions during the early months of life were successful. Infants who participated in motor-based care showed wider developmental gains in comparison to those who received the usual care [32], showing the significance of the early, intensive treatment programme.

### 3.4. Developmental Coordination Disorder

Developmental Coordination Disorder (DCD) is defined as significant motor coordination deficits that interfere with the effective engagement in daily activities [5]. Although DCD is more frequently detected in late childhood, there is positive evidence that early signs can be identified and are specific to prevent later sequelae. An early recognition scoping review [15] points to the necessity of monitoring motor development during early infancy and preschool years to identify patterns in pre-term or early childhood symptoms of coordination problems. Review articles and meta-analyses on motor interventions in children with DCD suggested that specific motor interventions are effective in ameliorating deficits in gross and fine motor skills and enhance functionality [5,15]. Successful programs often emphasize task-specific practice, repetitive performance of functional tasks, and provide feedback to support motor learning [33]. Interventions involving meaningful, real-world applications are important and useful in their daily life. There is the possibility that longer-term engagement limits may be lessened through early intervention. Children who get the support they need when required often show higher levels of confidence, engagement in physical activity, and attendance at school and social events. The above findings highlight the advantage of intervention strategies that are proactive in nature.

### 3.5. Autism Spectrum Disorder

Motor impairments are becoming a commonality among individuals with autism spectrum disorder (ASD). Several children with ASD have developmental delays in gross motor coordination, balance, and motor planning. While previous research considered motor impairment to be secondary, recent findings indicated that it may contribute substantially to daily functioning. Early motor skill training interventions for children with autism spectrum disorder, tested in a pilot trial, led to improvements in both motor performance and social engagement [17]. The above finding suggests that interventions targeting motor skills may offer benefits beyond physical improvements. Better motor skills can encourage children to participate more in play and social activities, creating additional opportunities for learning. However, there is less research on ASD compared to other groups, so more trials are needed to confirm these results and determine the best intervention strategies [17].

### 3.6. Congenital Medical Conditions

Children with congenital medical conditions, especially those with early surgeries, have been shown to be at a higher developmental risk due to complications associated with their diseases, prolonged hospitalization, and limited movement opportunities. Early motor interventions for infants with congenital heart disease following open-heart surgery were shown to provide some support and a mixed effect [34]. Research included in this review suggests more structured motor stimulation programs could positively impact developmental outcomes and motor progression. Nevertheless, the small number of studies, small sample sizes, and variability in intervention designs highlight the need for additional studies. For this group, standardized protocols and longer-term outcome measures would be important moving steps [34].

### 3.7. High-Risk and Environmentally Vulnerable Populations

Early motor interventions are also critical for children who are exposed to environmental risks such as poverty, low stimulation, or limited access to medical healthcare. Experimental evidence based on community programmes showed the benefits for cognitive and motor development of children in high-burden districts after engaging in structured early intervention programmes [3]. In a similar fashion, early childhood intervention has positive impact on motor development and general functioning. The current data indicate that early motor intervention programs are not limited to the medical setting and can be preventive in a child exposed to environmental adversity [33,35].

### 3.8. Typically Developing Children

Many studies on early motor intervention are designed for clinical cohorts. However, considerable evidence shows that typically developing children also benefit from early motor intervention. Despite a limited focus on early motor interventions for general development, meta-analyses of core fundamental movement skill programs showed significant improvements in motor competence in structured intervention programs [6,8,15,24]. Programs delivered in childcare centers, preschools, and kindergartens were especially effective. Crucially, motor competence of typically developing children was positively related to a range of developmental outcomes. Better motor competencies were also associated with higher cognitive capacity and better forms of social skills, which may imply that early motor programming provides protective effects even in the absence of rehabilitation [6,8,15,24].

### 3.9. Intervention Characteristics Associated with Effectiveness

Across populations and study designs many of the intervention characteristics consistently predicted positive developmental outcomes.

#### 3.9.1. Early Initiation

Interventions initiated in infancy or the early years of toddlerhood had stronger effects than those initiated in later childhood. The earlier the better, as time is used as an opportunity to seize on sensitive developmental windows when brain systems are most sensitive to experience [9,12,17,18,19,36].

Delayed intervention might squander opportunities to influence developmental trajectories.

#### 3.9.2. Active Participation

Research findings suggested that active participation and learning had a direct correlation with motor development. The activities that involve infants or learners in creating their own actions consistently led to better scores than those relying on passive stimulation programs [8,22,24].

#### 3.9.3. Task-Specific Practice

Training specific to a task—by which children repeatedly engage in meaningful, functional activities—is related to better outcomes for all populations. This approach facilitates daily life skill transfer and promotes functional independence [6,17,26,33,36].

#### 3.9.4. Intensity and Repetition

Sufficient dosage of intervention is mandatory. By introducing a repeated practice program for a relatively long enough time, programs that focus on repeated practice appear to have better and lasting outcomes compared to low-intensity interventions [31]. Insufficient practice may compromise the acquisition of skills and decrease the effectiveness of the intervention.

#### 3.9.5. Family Involvement

Caregiver active participation is a large component to success. Parent-mediated programs give children the opportunity to practice more, allow for skill transfer, and develop consistency across settings. Family involvement improves outcomes among many individuals, including preterm infants and children at developmental risk [13,19].

#### 3.9.6. Ecological Validity

Interventions carried out in natural environments, such as homes, childcare centers, and community settings, provide opportunities to engage in practical skills learning. Ecological approaches understand that development is not carried out in isolated clinic sessions but emerges naturally from daily contexts [6,24].

### 3.10. Cross-Population Patterns

Cross-population comparisons across all studies were observed. Despite differences in diagnosis, setting, and intervention models, several general trends have been observed across studies over time:

- early intervention over delayed intervention yields better results
- active practice outperforms passive stimulation
- goal-directed training improves functional outcomes
- family-centered methods improve effectiveness
- greater intensity yields stronger effects compared to lower intensity

These common threads indicate that the early motor interventions that are effective use fundamental concepts that span all diagnostic differences.

### 3.11. Developmental Cascade Effects

A prominent finding suggested improvements in motor competence may be achieved before improvements in other developmental domains. Motor interventions have also been related to improved attention, social engagement, participation, and autonomy. These results are consistent with developmental cascade models suggesting that alterations in one domain may impact other developmentally domains as well [14,17,18,23,26,28,30,36,37]. Early intervention may indirectly promote cognitive and social development by enhancing motor skills.

### 3.12. Emerging Technologies in Early Assessment and Intervention

Novel technology in early assessment and intervention appeared recently in literature. Recent studies showed that early detection of motor delay is becoming even more relevant due to digital advancements. A systematic review of portable assessment tools that included wearable sensors and motion-analysis technologies allowed the early detection of atypical motor patterns and the ability to tailor interventions [38]. While these emerging technologies remain in their infancy, they provide a means to augment early intervention services. Emerging technologies, including wearable sensors, motion tracking systems, and artificial intelligence–based assessment tools, offer new opportunities for early detection and personalized intervention. However, further research is needed to establish their clinical validity, accessibility, and cost-effectiveness.

### 3.13. Methodological Characteristics of the Evidence Base

A review of the study designs demonstrated multiple methodological approaches. Systematic reviews and meta-analyses provided significant evidence, indicating continuing interest in early motor intervention. Nonetheless, there are limited large randomized controlled trials and thus no causal inferences may be included. General methodological weaknesses between studies include small sample sizes, variability in intervention protocols; inconsistent outcome metrics; and limited long-term follow-up. These limitations hamper cross-study synthesis and reinforce the need for more rigorous designs.

## 4. Discussion

### 4.1. Background of Main Results

The present review synthesized findings from thirty studies examining early motor interventions across various pediatric populations, including preterm infants, children at risk of cerebral palsy, those with developmental coordination disorder, autism spectrum disorder, congenital medical conditions, and typically developing children. Evidence consistently supports that early motor interventions improve motor, cognitive, social, and functional outcomes. Interventions that start earlier are more intense, child-centered, and contextually responsive tend to lead to more positive developmental results. These benefits extend beyond motor skills to include cognitive and behavioral control [5,6]. The similar effects observed across different diagnoses suggest these interventions work through shared developmental pathways. Effective programs focus on system-level processes to enhance overall functioning, rather than targeting single deficits. Caregiver-mediated and home-based interventions also contribute to improved outcomes, emphasizing the importance of ecological, family-centered approaches. Although the findings across studies are generally consistent, important sources of heterogeneity should be considered. Variations in intervention intensity, duration, delivery setting, and outcome measures may partly explain differences in reported effectiveness. For example, studies employing high-intensity, task-specific interventions tended to report stronger outcomes compared to those using lower-intensity or less structured approaches. These findings reflect clear cross-study comparisons and highlight both consistencies and sources of heterogeneity across intervention approaches.

Furthermore, inconsistencies across studies may reflect differences in participant characteristics, such as baseline developmental status or comorbidities. These factors highlight the need for more standardized intervention protocols and outcome measures to improve comparability across studies.

### 4.2. Motor Development as a Driver of Development

Motor development is increasingly recognized as a key driver of broader developmental progress. Previously seen mainly as an indicator of neural maturation, motor skills now are understood as enabling children to explore, act, manipulate their environment, and communicate with others. These experiences provide sensory input that shapes neural circuits critical for cognitive learning [1]. There is substantial evidence linking both gross and fine motor skills with positive cognitive and social development in early childhood [3,6]. Structured motor programs provide essential foundations for physical competencies, participation, and healthy behaviors.

### 4.3. Timing of Intervention

Early intervention, beginning in infancy or toddlerhood, leads to greater and more enduring benefits than programs commencing later in life. This pattern holds true across populations, including preterm infants and children at risk for cerebral palsy [1,8,9,14,18,28,36]. Early intervention leverages heightened brain plasticity and enables better neural network reorganization. Current clinical guidelines recommend initiating therapy as soon as risk is identified rather than waiting for a formal diagnosis, as delays in treatment may cause harm [1,8,9,14,18,28,36].

### 4.4. Impact Mechanism of Intervention Effects

Early motor interventions affect development through multiple interacting mechanisms. Active movement promotes sensorimotor integration by linking motor activity with sensory feedback, strengthening neural pathways fundamental to motor learning [3,4]. Motor skill improvements increase opportunities for environmental exploration and social interaction, which likely contribute to gains observed in cognitive and social domains [10,14]. Task-specific, repetitive practice consolidates learning, and fosters transfer to daily life. Additionally, caregiver involvement supporting more frequent practice and reinforcement in everyday settings, further enhance outcomes [2,22].

### 4.5. Family-Centered and Ecological Approaches

Emerging evidence highlights the importance of family-centered and ecological intervention models. Unlike traditional therapist-led clinic programs, caregiver-driven approaches embed interventions within daily routines at home, childcare, and community settings, improving relevance and sustainability [13,19]. Culturally appropriate, play-based strategies may enhance motivation and engagement [13,19]. Empowering caregivers to independently implement interventions increases opportunities for practice in developmentally appropriate contexts.

### 4.6. Consistency Among Groups of Populations

The findings of the present review support a cross-population conceptual framework for early motor intervention, highlighting shared mechanisms that underpin effectiveness across diverse pediatric groups. Across diverse social and clinical groups, early motor interventions share several key elements linked to success: early initiation, active involvement, task specificity, sufficient intensity, and family participation. This convergence suggests that universal developmental principles underlie effective early motor interventions, supporting flexible cross-diagnostic models that improve clinical efficiency. Taken together, these findings reinforce the proposed framework, suggesting that early motor interventions operate through common developmental principles rather than diagnosis-specific mechanisms.

### 4.7. Implications for Prevention

Early motor interventions can benefit even typically developing children. Programs in preschool and childcare settings improve motor competence and foundational skills [6,23,24]. Given associations between motor skills and exercise, social interaction, and cognition, these interventions may promote long-term health and development, especially in children exposed to socioeconomic or environmental risks [22].

### 4.8. Emerging Role of Technology

New technologies like wearable devices and motion capture systems enhance early assessment by enabling more accurate and rapid detection of atypical motor development and tracking individual progress [38].

### 4.9. Methodological Aspects

Despite encouraging results, challenges persist. Many studies have small sample sizes limiting statistical power and generalizability. The wide variation in intervention protocols and outcome measures complicates cross-study comparisons [3,19].

### 4.10. Integration of Findings

Overall, early motor interventions should be viewed as comprehensive developmental processes promoting learning, participation, and autonomy rather than narrow therapeutic treatments. Clinicians and researchers need to recognize motor intervention as supporting multiple developmental domains, not only physical impairment. Studies assessing multidomain outcomes beyond motor metrics are essential.

## 5. Strengths and Limitations

### 5.1. Strengths

This scoping review provides a comprehensive overview of early motor intervention research across multiple pediatric populations, settings, and intervention approaches. It integrates findings from a wide range of study designs, including systematic reviews, meta-analyses, randomized controlled trials, pilot studies, and clinical guidelines [1,6,10,26,37,39].

A key strength of this review is its cross-population perspective, which enables the identification of common principles underlying effective intervention strategies. Despite heterogeneity in diagnoses and intervention models, consistent patterns were observed, including the importance of early initiation, active participation, task-specific practice, and caregiver involvement.

Additionally, the inclusion of multidomain outcomes—encompassing motor, cognitive, social, and participation-related domains—provides a more comprehensive understanding of the broader developmental impact of early motor interventions [1,3,5].

The review also incorporates emerging areas of research, such as the role of technology in early assessment and intervention, highlighting future directions for innovation in the field [8,38].

### 5.2. Limitations

This review has several limitations that should be considered when interpreting the findings.

- First, the study is based on predefined inclusion criteria and systematic database searches rather than a fully systematic database search, which may have resulted in the exclusion of relevant studies.
- Second, no formal risk-of-bias assessment was conducted, as the primary aim of this scoping review was to map the breadth of available evidence rather than to evaluate study quality.
- Third, substantial heterogeneity was observed across studies in terms of intervention type, intensity, duration, outcome measures, and follow-up periods, limiting direct comparisons and synthesis of findings [3,39].
- Furthermore, many included studies had relatively small sample sizes, which may affect the generalizability of results.
- Finally, the lack of long-term follow-up data in many studies limits conclusions regarding the sustainability of intervention effects over time.

## 6. Clinical Implications

- Early detection and referral: Focus on screening infants early for motor delays or developmental risk and initiate intervention during periods of high neuroplasticity [1,8,36,37].
- Active, task-specific practice: Emphasize interventions that actively engage children in meaningful, repetitive, task-relevant activities, as active motor engagement has been consistently associated with improved developmental outcomes [10,33,35,36].
- Family-centered approaches: Train caregivers to embed intervention strategies into daily routines and coach them to sustain practice activities, as caregiver-mediated interventions have been shown to enhance developmental gains and generalization of skills [7,13,19].
- Integration into natural environments: Deliver interventions in home, childcare, and community contexts to enhance functional relevance and sustainable progress [13,24].
- Multidomain monitoring: Track progress across motor, cognitive, and social domains to capture the broader impact of motor interventions [1,3,5].
- Despite promising evidence, several barriers may limit the implementation of early motor interventions in real-world settings. These include limited access to trained professionals, variability in healthcare resources, financial constraints, and differences in caregiver engagement. Cultural factors and health system infrastructure may also influence the feasibility and effectiveness of intervention delivery.

## 7. Research Priorities

- Future research should prioritize the standardization of intervention reporting, including dosage, frequency, intensity, and duration, as variability in these parameters limits comparability across studies [3,39].
- There is a need for larger, multicenter randomized controlled trials with rigorous methodological design to strengthen causal inference and improve the overall quality of evidence [9,37].
- Long-term follow-up studies are essential to determine whether early intervention effects are sustained over time and translate into functional, academic, and participation outcomes later in childhood [31,36].
- Future studies should also aim to develop and evaluate flexible, cross-diagnostic intervention models that can be applied across diverse pediatric populations, reflecting shared developmental mechanisms [5,14].
- The role of emerging digital technologies in early assessment and intervention planning should be further explored, particularly in terms of clinical validity, accessibility, and cost-effectiveness [8,38].
- Finally, research should address cost-effectiveness and implementation strategies to support integration of early motor interventions into routine clinical practice and public health systems.

## 8. Conclusions

This scoping review supports the effectiveness of early motor interventions across diverse pediatric populations. Successful programs consistently incorporate early initiation, active participation, task-specific practice, sufficient intensity, and caregiver involvement. These shared characteristics highlight common underlying mechanisms that drive developmental change across diagnostic groups.

Benefits extend beyond motor development to cognitive, social, and participation-related outcomes, reinforcing the central role of motor competence in early development [1,3,5]. The consistency of findings across populations suggests that early motor interventions operate through shared developmental pathways rather than diagnosis-specific mechanisms.

Although methodological variability and limited long-term follow-up restrict definitive conclusions, the overall evidence base strongly supports early, active, and family-centered approaches. Future research should prioritize standardized intervention protocols, high-quality randomized controlled trials, and long-term outcome tracking.

In conclusion, early motor interventions demonstrate consistent benefits across multiple developmental domains and pediatric populations. However, variability in study design and limited long-term evidence highlight the need for more rigorous research. Future studies should prioritize high-quality randomized controlled trials, standardized intervention protocols, and long-term follow-up to strengthen the evidence base and support clinical decision-making.

## Data Availability

No new data were created.

## Supplementary Materials

The following supporting information can be downloaded at: https://www.mdpi.com/article/10.3390/children13050644/s1, Table S1: Detailed Characteristics of Included Studies.

## References

[1] Capio C.M., Mendoza N.B., Jones R.A., Masters R.S., Lee K. (2024). The contributions of motor skill proficiency to cognitive and social development in early childhood. Sci. Rep..

[2] Veldman S.L., Santos R., Jones R.A., Sousa-Sá E., Okely A.D. (2019). Associations between gross motor skills and cognitive development in toddlers. Early Hum. Dev..

[3] Shi P., Feng X. (2022). Motor Skills and Cognitive Benefits in Children and Adolescents: Relationship, Mechanism and Perspectives. Front. Psychol..

[4] Gandotra A., Csaba S., Sattar Y., Cserényi V., Bizonics R., Cserjesi R., Kotyuk E. (2022). A Meta-analysis of the Relationship between Motor Skills and Executive Functions in Typically-developing Children. J. Cogn. Dev..

[5] Smits-Engelsman B., Vinçon S., Blank R., Quadrado V.H., Polatajko H., Wilson P.H. (2018). Evaluating the evidence for motor-based interventions in developmental coordination disorder: A systematic review and meta-analysis. Res. Dev. Disabil..

[6] Zhang D., Li F., Liang W., Xu S., Yang Y. (2024). Effect of Intervention Programs to Promote Fundamental Motor Skills among Typically Developing Children: A Systematic Review and Meta-analysis. Child. Youth Serv. Rev..

[7] Damiano D.L., Longo E. (2022). Early Intervention for Children with Cerebral Palsy: A Systematic Review and Meta-Analysis. Int. J. Environ. Res. Public Health.

[8] Morgan C., Darrah J., Gordon A.M., Harbourne R., Spittle A., Johnson R., Fetters L. (2016). Effectiveness of motor interventions in infants with cerebral palsy: A systematic review. Dev. Med. Child. Neurol..

[9] Damiano D.L., Longo E. (2021). Early intervention evidence for infants with or at risk for cerebral palsy: An overview of systematic reviews. Dev. Med. Child. Neurol..

[10] Zeng N., Ayyub M., Sun H., Wen X., Xiang P., Gao Z. (2017). Effects of Physical Activity on Motor Skills and Cognitive Development in Early Childhood: A Systematic Review. BioMed. Res. Int..

[11] Morgan C., Novak I., Badawi N. (2016). Effectiveness of motor interventions in infants with cerebral palsy: A systematic review. Dev. Med. Child. Neurol..

[12] Bozgan-Baş D., Yardimci-Lokmanoğlu B.N., Mutlu A. (2025). The Impact of Early Intervention on Early Spontaneous Movements of Infants: A Systematic Review. Early Hum. Dev..

[13] Bernabe-Zuñiga J.E., Rodriguez-Lucenilla M.I., Alias-Castillo A.J., Rueda-Ruzafa L., Roman P., del Mar Sanchez-Joya M. (2025). Early interventions with parental participation and their implications on the neurodevelopment of premature children: A systematic review and meta-analysis. Eur. Child. Adolesc. Psychiatry.

[14] Blauw-Hospers C.H., de Graaf-Peters V.B., Dirks T., Bos A.F., Hadders-Algra M. (2007). Does early intervention in infants at high risk for a developmental motor disorder improve motor and cognitive development?. Neurosci. Biobehav. Rev..

[15] Lee E.J., Zwicker J.G. (2021). Early identification of children with/at risk of developmental coordination disorder: A scoping review. Dev. Med. Child. Neurol..

[16] Kaeslin R., Latal B., Mitteregger E. (2023). A systematic review of early motor interventions for infants with congenital heart disease and open-heart surgery. Syst. Rev..

[17] Ketcheson L., Hauck J., Ulrich D. (2017). The effects of an early motor skill intervention on motor skills, levels of physical activity, and socialization in young children with autism spectrum disorder: A pilot study. Autism Int. J. Res. Pract..

[18] Orton J., Spittle A., Doyle L.W., Anderson P.J. (2024). Early developmental intervention programmes provided post hospital discharge to prevent motor and cognitive impairment in preterm infants. Cochrane Database Syst. Rev..

[19] Sayed R.M., Ahmed G.S., Abd El-Maksoud G.M. (2025). The Effectiveness of an Early Intervention Program on the Development of Motor Functions in Children with Cerebral Palsy Study on the Home Environment. J. Environ. Sci..

[20] Peters M.D., Godfrey C.M., Khalil H., McInerney P., Parker D., Soares C.B. (2015). Guidance for conducting systematic scoping reviews. Int. J. Evid.-Based Healthc..

[21] Tricco A.C., Lillie E., Zarin W., O’Brien K.K., Colquhoun H., Levac D., Moher D., Peters M.D.J., Horsley T., Weeks L. (2018). PRISMA Extension for Scoping Reviews (PRISMA-ScR): Checklist and Explanation. Ann. Intern. Med..

[22] Suryavanshi P., Srivastava S. (2025). The Effect of Early Intervention Programme on Mental and Motor Development of Children in Anganwadi Centres of High Burden Districts of U.P., India. Int. J. Home Sci..

[23] van der Walt J., Plastow N.A., Unger M. (2020). Motor skill intervention for pre-school children: A scoping review. Afr. J. Disabil..

[24] Wick K., Leeger-Aschmann C.S., Monn N.D., Radtke T., Ott L.V., Rebholz C.E., Cruz S., Gerber N., Schmutz E.A., Puder J.J. (2017). Interventions to Promote Fundamental Movement Skills in Childcare and Kindergarten: A Systematic Review and Meta-Analysis. Sports Med..

[25] Badura A., Fiander M., Gabriel A., Pirinu M., Beccani L., Soll R.F., Cracknell J., Faas D., Wellmann S., Bruschettini M. (2025). In-hospital motor interventions to reduce neurodevelopmental impairment in preterm infants: A scoping review. Cochrane Database Syst. Rev..

[26] Orton J., Doyle L.W., Tripathi T., Boyd R., Anderson P.J., Spittle A., Cochrane Neonatal Group (2015). Early developmental intervention programmes provided post hospital discharge to prevent motor and cognitive impairment in preterm infants. Cochrane Database Syst. Rev..

[27] De Bruyn N., Hanssen B., Mailleux L., Van den Broeck C., Samijn B. (2025). Early Intervention Including an Active Motor Component in Preterms with Varying Risks for Neuromotor Delay: A Systematic Review and Narrative Synthesis. J. Clin. Med..

[28] Orton J., Spittle A., Doyle L., Anderson P., Boyd R. (2009). Do Early Intervention Programmes Improve Cognitive and Motor Outcomes for Preterm Infants After Discharge? A Systematic Review. Dev. Med. Child. Neurol..

[29] Dumuids-Vernet M.-V., Forma V., Provasi J., Anderson D.I., Hinnekens E., Soyez E., Strassel M., Guéret L., Hym C., Huet V. (2023). Stimulating the motor development of very premature infants: Effects of early crawling training on a mini-skateboard. Front. Pediatr..

[30] Hutchon B., Gibbs D., Harniess P., Jary S., Crossley S.-L., Moffat J.V., Basu N., Basu A.P. (2019). Early intervention programmes for infants at high risk of atypical neurodevelopmental outcome. Dev. Med. Child. Neurol..

[31] Morgan C., Fetters L., Adde L., Badawi N., Bancale A., Boyd R.N., Chorna O., Cioni G., Damiano D.L., Darrah J. (2021). Early Intervention for Children Aged 0 to 2 Years with or at High Risk of Cerebral Palsy: International Clinical Practice Guideline Based on Systematic Reviews. JAMA Pediatr..

[32] Harbourne R.T., Willett S., Kyvelidou A., Deffeyes J., Stergiou N. (2020). A Comparison of Two Physical Therapy Interventions for Postural Control in Infants with Cerebral Palsy: A Randomized Clinical Trial. Phys. Ther..

[33] Logan S.W., Robinson L.E., Wilson A.E., Lucas W.A. (2012). Getting the fundamentals of movement: A meta-analysis of the effectiveness of motor skill interventions in children. Child. Care Health Dev..

[34] Tabosa T.A., Silva A.Z., Nobre G.C. (2022). MINI-REVIEW: Contribution of Early Intervention Models to Child Motor Development. Braz. J. Mot. Behav..

[35] Larasati S.A., Sit M. (2025). Managing Traditional ‘Cat and Mouse’ Play to Optimize Gross Motor Development in Early Childhood. J. Educ. Manag. Res..

[36] Dumuids-Vernet M.V., Provasi J., Anderson D.I., Barbu-Roth M. (2022). Effects of Early Motor Interventions on Gross Motor and Locomotor Development for Infants at-Risk of Motor Delay: A Systematic Review. Front. Pediatr..

[37] Yildiz A., Yildiz R., Apaydin U., Efkere P.A., Gücüyener K., Hirfanoglu I.M., Elbasan B. (2025). Effects of SAFE Early Intervention Approach in the First Months of Life in Infants at Risk: A Randomized Controlled Study. Child. Care Health Dev..

[38] Deng W., Li J., Zhang X., Brown S., Miller K. (2025). A systematic review of portable technologies for the early assessment of motor development in infants. npj Digit. Med..

[39] Blauw-Hospers C.H., Hadders-Algra M. (2005). A systematic review of the effects of early intervention on motor development. Dev. Med. Child. Neurol..

