# Supplementary material for: Early Motor Interventions in Infants and Young Children: A Comprehensive Scoping Review
Source: Children (Basel). 2026 May 4;13(5):644. doi: 10.3390/children13050644 (PMC13204060; doi:10.3390/children13050644)
Supplement: Supplementary file 1 [file children-13-00644-s001.zip › children-4265242-supplementary.pdf]

Supplementary Table S1. Detailed Characteristics of Included Studies

**Author (Year)**	**Study Design**	**Population**	**Intervention Type**	**Outcomes Measured**	**Key Findings**
[1]	Observational	Typically developing	Motor programs	Cognitive/social	Positive
[5]	Systematic review	DCD	Motor intervention	Motor	Improved
[6]	Systematic review	Typically developing	FMS	Motor	Positive
[9]	Systematic review	CP risk	Therapeutic intervention	Motor	Effective
[11]	Systematic review	CP	Motor intervention	Motor	Effective
[13]	Systematic review & meta-analysis	Preterm infants	Parent-mediated intervention	Neurodevelopment	Improved outcomes
[14]	Systematic review	Infants at risk	Developmental programs	Motor	Moderate effects
[39]	Systematic review	Infants at risk	Early intervention	Motor/cognitive	Improved

[34]	Mini-review	Children	Models	Motor	Positive
[18]	Systematic review	Preterm	Post-discharge	Motor/cognitive	Reduced risk
[15]	Scoping review	DCD risk	Identification	Motor	Important
[17]	Pilot	ASD	Motor training	Motor/social	Improved
[16]	Systematic review	CHD	Motor programs	Motor	Limited evidence
[19]	Intervention	CP	Home-based	Motor	Improved
[22]	Intervention	High-risk	Community	Motor/cognitive	Improved
[23]	Scoping review	Preschool	Motor programs	Motor	Supportive
[24]	Systematic review	Children	FMS programs	Motor	Strong
[25]	Scoping review	Preterm infants	In-hospital motor intervention	Neurodevelopment	Reduced impairment
[26]	Systematic review	Preterm	Early programs	Motor/cognitive	Moderate

[27]	Systematic review	Preterm	Motor intervention	Motor	Benefits
[28]	RCT	Preterm infants	Early developmental intervention	Motor & cognitive	Improved outcomes
[29]	Experimental	Preterm	Crawling training	Motor	Effective
[30]	Systematic review	High-risk	Early intervention	Development	Improved
[31]	Guideline	CP risk	Guideline intervention	Motor	Strong
[32]	RCT	Infants with neuromotor disorders	START-Play	Motor & cognitive	Improved outcomes
[33]	Meta-analysis	Children	Motor skills	Motor	Significant
[35]	Intervention	Typically developing	Play-based	Motor	Improved
[36]	Systematic review	At-risk infants	Motor intervention	Motor	Improved
[37]	RCT	Infants	SAFE intervention	Development	Improved

[38]	Systematic review	Infants	Tech assessment	Motor	Promising
